# Urothelial Senescence in the Pathophysiology of Diabetic Bladder Dysfunction—A Novel Hypothesis

**DOI:** 10.3389/fsurg.2018.00072

**Published:** 2018-12-04

**Authors:** Nicole S. Klee, Cameron G. McCarthy, Steven Lewis, Jaine L. McKenzie, Julie E. Vincent, R. Clinton Webb

**Affiliations:** ^1^Department of Physiology, Medical College of Georgia at Augusta University, Augusta, GA, United States; ^2^Department of Physiology and Pharmacology, University of Toledo College of Medicine and Life Sciences, Toledo, OH, United States; ^3^Department of Surgery, Medical College of Georgia at Augusta University, Augusta, GA, United States

**Keywords:** lower urinary tract, senescence, urothelium, diabetic cystopathy, diabetic bladder

## Abstract

Diabetic bladder dysfunction (DBD) is a well-recognized and common symptom affecting up to 50% of all diabetic patients. DBD has a broad range of clinical presentations ranging from overactive to underactive bladder symptoms that develops in middle-aged to elderly patients with long standing and poorly controlled diabetes. Low efficacy of current therapeutics and lifestyle interventions combined with high national healthcare costs highlight the need for more research into bladder dysfunction pathophysiology and novel treatment options. Cellular senescence is an age-related physiologic process in which cells undergo irreversible growth arrest induced by replicative exhaustion and damaging insults. While controlled senescence negatively regulates cell proliferation and promotes tissue regeneration, uncontrolled senescence is known to result in tissue dysfunction through enhanced secretion of inflammatory factors. This review presents previous scientific findings and current hypotheses that characterize diabetic bladder dysfunction. Further, we propose the novel hypothesis that cellular senescence within the urothelial layer of the bladder contributes to the pro-inflammatory/pro-oxidant environment and symptoms of diabetic bladder dysfunction. Our results show increased cellular senescence in the urothelial layer of the bladder; however, whether this phenomenon is the cause or effect of DBD is unknown. The urothelial layer of the bladder is made up of transitional epithelia specialized to contract and expand with demand and plays an active role in transmission by modulating afferent activity. Transition from normal functioning urothelial cells to secretory senescence cells would not only disrupt the barrier function of this layer but may result in altered signaling and sensation of bladder fullness; dysfunction of this layer is known to result in symptoms of frequency and urgency. Future DBD therapeutics may benefit from targeting and preventing early transition of urothelial cells to senescent cells.

## Introduction

The bladder functions to store and excrete urine, which is required to remove waste from the body. Normal urine storage occurs over a broad range of volumes that empties in a controlled and efficient manner at an appropriate time and location. This not only requires accurate sensation of bladder fullness, but also coordinated actions of the parasympathetic and sympathetic nervous systems to contract and relax the urinary bladder wall and urethral sphincters (1). Dysregulation of the urinary bladder results in lower urinary tract symptoms (LUTS) such as nocturia, urgency, frequency, incontinence, etc. (2), which if left untreated, can lead to systemic inflammatory response syndrome and sepsis, and even possibly death (3, 4). LUTS have a significant negative impact on quality of life and are strongly associated with depression (5–7). These symptoms also impact United States health care costs; overactive bladder alone is estimated to cost $82.6 billion by 2020 (8). LUTS are frequent in the general population, affecting 62.5% of men and 66.6% of women (9). The incidence of LUTS also increases with age (10) and with an aging population (11), we expect the number of patients experiencing LUTS to be on the rise.

**Figure 1:**
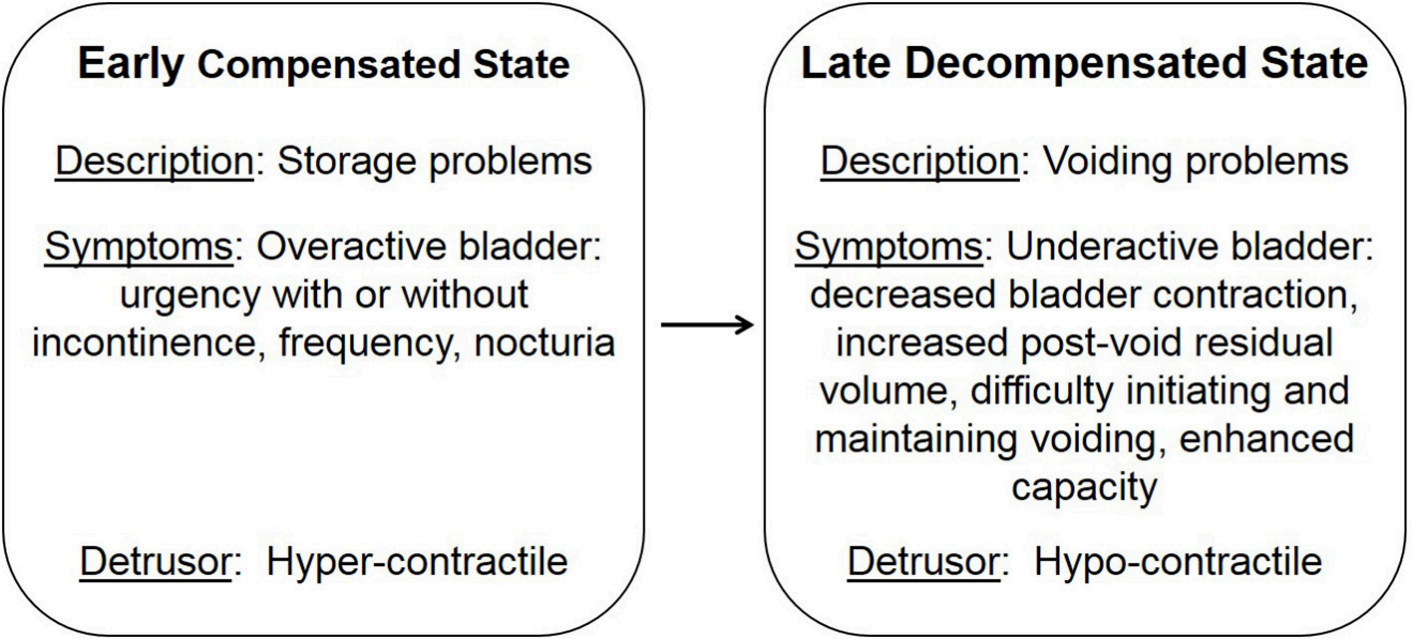
Temporal progression of diabetic bladder dysfunction. Adapted from Daneshgari et al. (13).

LUTS are not only associated with age, they are also comorbid with stroke, brain tumors, cerebral palsy, dementia, sexual dysfunction, spinal cord injury, multiple sclerosis, and diabetes (12–20). Diabetes is characterized by defects in the secretion or signaling of insulin that results in the impairment of glucose uptake and ensuing high plasma glucose levels (21). Initially, hyperglycemia results in an elevated concentration of glucose in the urine, exceeding the amount reabsorbed by the kidney, leading to osmotic diuresis and polyuria. During this early compensated state, patients may present with symptoms of overactive bladder, including urgency, frequency, and nocturia. Over time, the early compensated state progresses into a late decompensated state that research suggests is due to chronically high levels of glucose and oxidative stress (Figure 1; 22; 13). Patients within the late decompensated state present with symptoms of underactive bladder, including decreased bladder contraction, increased post-void residual volume, difficulty initiating and maintaining voiding, and enhanced capacity.

LUTS affect up to 50% of all diabetic patients and are referred to as diabetic bladder dysfunction (DBD) (13, 22). Despite recent advances in understanding DBD, underlying mechanisms are unclear and to complicate things further, there are few studies that parcel out pure age-dependent changes from dysfunction due to underlying pathology or disease (10, 23). Here, we discuss the current theories underlying DBD and a novel hypothesis that increased cellular senescence within the bladder is the initiating factor in tissue dysfunction, which culminates in symptoms of DBD.

## Theories and Hypotheses on the Etiology of Diabetic Bladder Dysfunction

**Figure 2:**
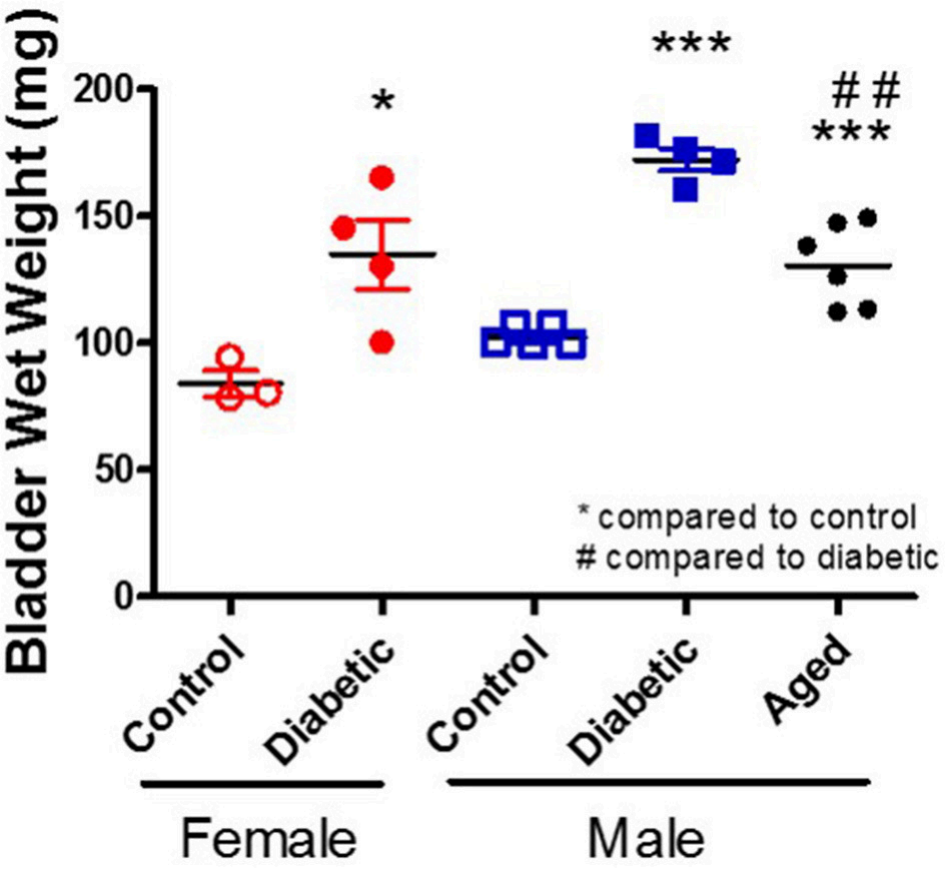
Increased bladder weight in diabetic and aged animals. Bladder wet weight was measured in female and male control and diabetic animals (18–20 w of age; 1 month post-STZ injections) and in aged male animals (53 w of age). Diabetes was induced using the combination of a high fat diet and one streptozotocin injection (30 mg/kg) in Wistar animals.

There are multiple suggested mechanisms underlying DBD symptoms that are generalized to the whole bladder although the majority are focused on the smooth muscle layer. Literature reports alterations within the components of muscle contraction (calcium handling, receptors, channels, protein isoforms, thin, and thick filaments) (24–29), oxidative stress (30, 31), inflammation (32–34), neuropathy (35–37), and age-related changes (38). Possibly the most consistently reported change is an increase in whole bladder weight (Figure 2; original data). Interestingly, diabetic animals and control aged animals have similar increases in bladder weight compared to young controls; these changes parallel the increased presence of LUTS with diabetes and age in human populations.

DBD encompasses both storage and voiding symptoms and has been hypothesized, by Daneshgari's group in 2009, to progress temporally from an early compensated state, where the bladder adapts to increased polyuria, to a late decompensated state due to chronically high levels of glucose and oxidative stress (13, 39, 40). This temporal hypothesis is the most current hypothesis detailing disease progression. In 2014, Chancellor further hypothesized that the early compensated state leads to the late decompensated state through tissue fatigue and changes in the structure and function of the bladder leading to ischemia, inflammation, oxidative stress, and this induces impaired contractility in the decompensated state (38).

This section highlights reported changes within the different layers of the urinary bladder. Scientific studies mentioned within this section rarely parcel out voiding behavior as an output measurement when investigating mechanisms of bladder dysfunction; therefore, classification into overactive or underactive bladder pathophysiology is unclear.

### Urinary Bladder

#### Urothelium

The urothelial layer is the inner most layer of the bladder that functions as a permeability barrier to urine, protecting the underlying tissue. It has sensory functions that respond to chemical, mechanical, and thermal stimuli by releasing factors such as ATP, acetylcholine, and nitric oxide to modulate afferent transmission, relaying the extent of bladder fullness (41–44). The urothelium is made up of three layers of cells: basal, intermediate, and apical or superficial cells (45). The superficial cells are the main component in barrier function and exhibit specialized tight junctions, which facilitate the prevention of unregulated chemical diffusion (45). Barrier dysfunction results in symptoms such as urgency and frequency and this layer has been shown to be altered by diabetes (46). For example, Hanna-Mitchell et al. in a streptozotocin (STZ; 65 mg/kg)—induced model of diabetes, through preferential toxicity to the insulin producing β-cells of the pancreas, reported that exposure to chronic hyperglycemia induces desquamation of the superficial cells, subsequently contributing to breaches in the barrier function of the urothelium (47). At a later time point, the urothelium exhibited barrier repair and vast changes both in cell morphology and gene expression (glucose metabolism, cell survival and proliferation, parasympathetic receptors, and cell stress/death), which are also factors that are altered in senescence (47). Further, Wang et al. reported increased urothelial inflammation in overactive bladder, which they noted did not differ between overactive patients with or without diabetes; however, overactive bladder was diagnosed solely based on urgency and urgency incontinence (48). Initial diabetes-induced damage to the urothelial layer could contribute to symptoms of overactive bladder, whereas repair accompanied with cellular changes may indicate the transition between the compensated and decompensated states of DBD.

#### Lamina Propria

The lamina propria is a loose areolar connective tissue that is located deep to the urothelial layer. The lamina propria connects the urothelial layer to the underlying smooth muscle layer and is both blood perfused and innervated, supporting the urothelial layer with nutrients and neuromediators. This layer contains collagen as well as several cell types, including interstitial cells (IC), fibroblasts, and adipocytes (41). Studies have reported changes in collagen composition (both increased and decreased) possibly contributing to altered strength and stiffness of the tissue (40). Increased collagen levels within the lamina propria could lead to increased tissue stiffness and strength as well as decreased nutrient diffusion to the urothelial layer and initially contribute to symptoms of overactive bladder. Decreased collagen levels would result in decreased tissue and contribute to symptoms of underactive bladder. The function of resident cells of the lamina propria could also be affected by diabetes. Recent identification of IC seem to serve similar roles as IC of Cajal (ICC) in the gastrointestinal (GI) tract by regulating smooth muscle excitability and mediating responses to neurotransmitters (49–51). ICC have spontaneous pacemaker activity which is conducted to the smooth muscle layer through gap junctions resulting in electrical slow waves and phasic contractions. Neural inputs, from motor neurons in close proximity to ICC, can also induce contraction by electrically coupling with smooth muscle cells. This complex operates below the level of neural and hormonal smooth muscle regulation and a loss of any component or connectivity of the complex has been associated with GI motor disorders (52). For example, a decrease in the number of GI ICC is believed to contribute to disturbed GI motility in human patients with type 2 diabetes (53) and diabetic db/db mice (54). IC within the urinary bladder has gained much interest due to evidence of its association with bladder pathophysiology where the number of IC have been shown to be both increased (55, 56) and decreased in overactive bladder and bladder outlet obstruction (57). Further, alterations or disruptions of these IC have been proposed to disrupt homeostatic tissue function within the urinary bladder (55, 58, 59). Increases in the number of IC could in theory increase the amount of spontaneous smooth muscle activity; therefore contribute to symptoms of urgency within overactive bladder. In contrast, a decrease in the number of IC could lead to decreased contractility and symptoms present within underactive bladder.

#### Smooth Muscle

The smooth muscle layer of the urinary bladder functions to contract (parasympathetic nervous system) and relax (sympathetic nervous system) the bladder during micturition and urine storage, respectively. There are a variety of DBD studies focused at the layer of the smooth muscle that depict modifications within contractility, neurotransmitter release, receptor and channel expression, thick and thin filament and contractile protein expression, and protein phosphorylation; however, results are varied showing both increased and decreased expression of components mentioned (13, 25, 27–29, 31, 60).

### Urethra

The urethra is a duct that transfers urine from the urinary bladder to the exterior of the body. The urethra is held closed by two sphincters, the internal urethral sphincter, and the external urethral sphincter, which are made up of smooth and skeletal muscle, respectively. Alterations with urethral function have been reported, showing both smooth and skeletal muscle dysfunction of urethral relaxation during the voiding reflex (61, 62). This could contribute to increased bladder pressure, increased void volume, and trouble initiating and maintaining voiding, altering normal bladder function and causing further damage to the urinary bladder. Consequences of these alterations include increased intravesicle pressure, as well as increased detrusor muscle contraction as a compensatory mechanism, contributing to underactive bladder symptoms.

## Diabetes Is Associated With Increased Cellular Senescence

**Figure 3:**
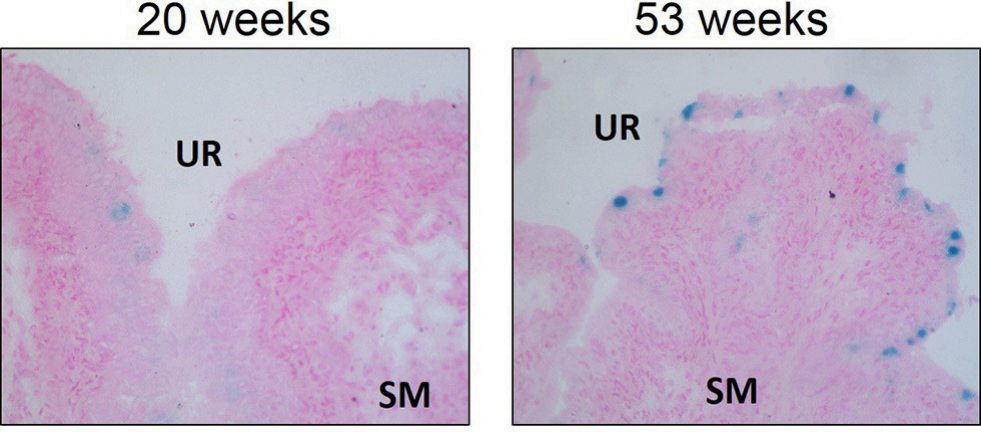
Senescence associated-β-galactosidase staining is increased in aged Wistar urothelium compared to young control counterparts. Senescence associated-β-galactosidase staining (blue) and nuclear fast red counterstain in young (20 w of age) animals and old (53 w of age) animals (left and right panels, respectively). Representative images of bladder SA-β-galactosidase at 200X. SM, smooth muscle. UR, urothelium.

Cellular senescence is a physiologic process in which replication-competent cells undergo irreversible cell cycle arrest induced essentially by any insult that produces DNA damage (replicative exhaustion and telomere shortening, excessive/ prolonged cellular stress) that can be grouped into three different subtypes: replicative senescence, stress-induced premature senescence, and oncogene-induced senescence (63, 64). This DNA damage results in the downstream activation of p53 and convergence on the inhibition of cyclin-dependent kinases (CDK), a family of protein kinases that are involved with cell cycle regulation (63). The inhibition of CDK–cyclin complexes results in proliferative arrest and the crucial component responsible for the implementation of senescence is the hypo-phosphorylated form of retinoblastoma protein (64). Senescence is generally considered a physiologic process and a protective mechanism to prevent morbidity by reducing the transmission of genomic defects to the next generation (63). In fact, studies suggest that controlled senescence promotes tissue regeneration via the recruitment of phagocytic immune cells to sites of injury after which senescent cells are then cleared (65, 66). Recruitment of immune cells depend on senescent secretory activity known as the senescence-associated secretory phenotype (SASP), allowing the secretion of pro-inflammatory cytokines and chemokines, reactive oxygen species (ROS), growth factors, and proteases into the local tissue microenvironment through autocrine and paracrine signaling (64, 67). An accumulation of senescent cells occurs with aging and is associated with tissue dysfunction, as well as numerous pathologies. Interestingly, we have evidence that urothelial senescence also increases with age (Figure 3; original data). Mechanistically, senescence-induced tissue dysfunction and pathology is thought to occur through the chronic release of SASP mediators and a heightened inflammatory state (64, 68–72).

**Figure 4:**
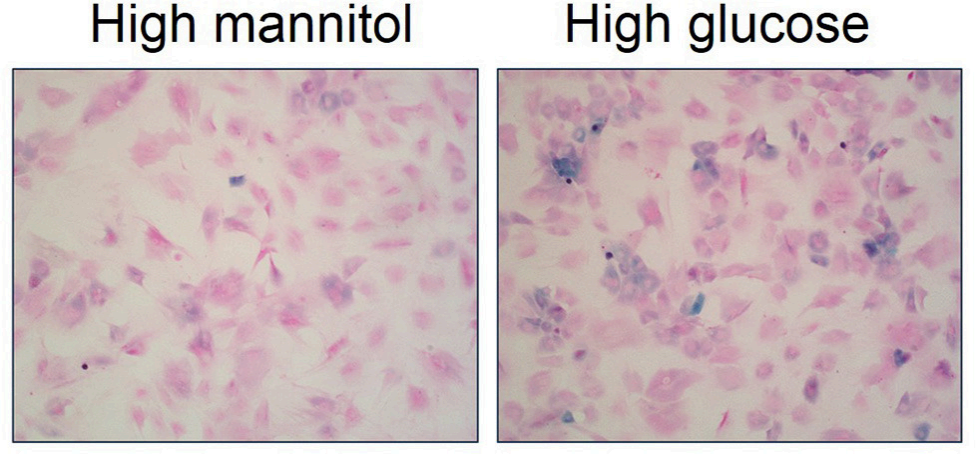
High glucose increases senescence associated-β-galactosidase staining in primary bladder smooth muscle cells. Senescence associated-β-galactosidase (blue) and nuclear fast red counterstain in high mannitol osmotic control (22 mM; left panel) and high glucose (22 mM; right panel) after a 4 day incubation. Representative images of primary bladder smooth muscle cell senescence associated-β-galactosidase at 100X.

Recent evidence suggests that cellular senescence is an important developmental contributor and/or consequence of type 2 diabetes (70). For example, several studies describe augmented inflammation and increased number of senescent cells within pancreatic β-cells of type 2 diabetic patients (73, 74). Monocytes from type 2 diabetic patients exhibit telomere shortening and increased oxidative DNA damage compared to healthy individuals (75). Fibroblasts isolated from diabetic wounds exhibit disturbed proliferation (76). Senescent cells are increased in adipose tissue of both type 2 diabetic animal models and patients (77, 78). Further, high glucose induces cellular senescence in multiple cell types *in vitro* including endothelial cells, mesothelial cells, vascular smooth muscle cells, mesenchymal stem cells, and skin fibroblasts (79–83). We also have evidence that high glucose increases cellular senescence in primary bladder smooth muscle cells (Figure 4; original data). Overall, evidence suggests an association between type 2 diabetes and increased cellular senescence, possibly via heightened (mitochondrial) oxidative stress (77, 78). In fact, targeting senescent cells in type 2 diabetes has been suggested as a therapeutic option (70). Low-grade systemic inflammation is a common manifestation of diabetes; however, the exact mechanisms that initiate this pathophysiological response, thereby contributing to diabetic complications, including DBD and LUTS is not known.

## The Immune Response and Inflammation in Diabetic Bladders

The urinary tract has several lines of defense that protect against infection. The first line of defense are superficial urothelial cells that not only act as a physical barrier, but also secrete pro-inflammatory mediators and antibacterial agents. Urothelial cells are connected by tight junctions, made up of 4–6 intracellular protein strands containing claudin, occludin, junction adhesion molecules, and zonula occludens that link these strands to the intracellular cytoskeleton (84, 85). This robust architecture is essential, and it contributes to the sterility of the bladder by preventing passive movement of particles between the blood and the urine. Further, these cells contain thick scallop-shaped plaques along the apical membrane that are covered with a sulfated polysaccharide glycosaminoglycan layer to further maintain the physical barrier of the bladder by deterring microbial colonization (84, 86, 87). In addition, urothelial cells also secrete factors that inhibit bacterial growth by interfering with the structure of the bacterial cell membrane or preventing protein synthesis within the cell (88–92).

Urothelial cells possess an abundance of pattern recognition receptors (PRRs) of the innate immune system including Toll-like receptors (TLRs), which recognize early indications of infection and release pro-inflammatory mediators (93). TLR4 is perhaps the best understood and most prevalent TLR in the urothelium; stimulation of which has been shown to result in expulsion of bacteria containing vacuoles from urothelial cells (94). Activation of TLR4 leads to interaction of its intracellular binding domain with an adaptor protein complex that includes MyD88 ultimately promoting the NF-κB pathway and transcription of cytokines such as interleukin 6 (IL-6) and interleukin 8 (IL-8) (94). TLR4 and its downstream mediators have been shown to be increased in DBD suggesting increased inflammation may play a role in DBD pathogenesis (34).

Bacterial infection can also activate caspase induced apoptosis of infected urothelial cells, effectively releasing contents into the lumen of the bladder. This not only removes bacteria, but it also attenuates excessive release of pro-inflammatory molecules. However, excessive removal of urothelial cells can expose underlying cell layers to toxins that are present within urine. As a protective measure, the urothelium secretes sonic hedgehog (SHH) to activate the WNT pathway, initiating urothelial cell proliferation to restore the epithelial barrier (95). This mechanism is reinforced by studies that show higher levels of apoptotic cells, antiproliferative factor, ATP, and vascular endothelial growth factor in the urine or tissue of patients with bladder infection (96–100). While the WNT pathway has not been studied in DBD, bladder thickness of the urothelium are increased in DBD and could be the result of this signaling process (32, 101).

Neutrophils are the first immune cells recruited to the urothelium from the blood vessels to the lumen of the bladder where they can act upon bacteria (102). The number of neutrophils recruited is congruent with the relative amount of bacteria present, and peaks approximately 6 h after activation (103). While neutrophils are highly effective at eliminating bacteria, they are also toxic to bladder tissue because they release ROS, increase expression of cyclooxygenase 2 (COX2), and induce expression of other cytotoxic products that cause inflammatory damage to the bladder. Neutrophil byproducts have been shown to cause hyperplasia and also predispose the bladder to persistent infections (104).

Bladder mast cells reside beneath the epithelium and are triggered via secretions of damaged or stressed epithelial cells in response to ATP, IL-33, and β-defensin (105–107). Mast cells regulate the immune response by releasing pro-inflammatory molecules such as histamine and TNF-α, which further contributes to the recruitment of neutrophils (108). Diabetic patients have significantly higher levels of mast cells in the bladder than patients without diabetes (48). Interestingly, TNF-α is not only elevated in bladder smooth muscle cells and serum isolated from diabetic mice, it also plays a critically important role in DBD by activating Rho kinase and ultimately causing bladder smooth muscle cell hypercontractility; neutralizing TNF-α improved bladder function (33).

## Oxidative Stress in Diabetic Bladders

Oxidative stress due to elevated levels of ROS has also been implicated in the pathogenesis of DBD. Diabetic hyperglycemia is capable of causing oxidative stress through several key mechanisms: upregulation of the polyol pathway, increased production of advanced glycation end-products, increased activity of protein kinase C, increased activation of the hexosamine pathway, and ultimately the overproduction of superoxide by the electron transport chain (109–115). As oxidative stress ensues, cellular functions are impaired due to damaged DNA, mitochondrial enzyme dysfunction, and phospholipid bilayer destruction. These damaging effects contribute to morphological and functional changes associated with DBD.

Multiple studies show the presence of oxidative stress and subsequent structural and functional modifications in diabetic bladders. For example, bladders from type 2 diabetic female Sprague-Dawley rats exhibit significant increases in nitrotyrosine and manganese superoxide dismutase protein expression compared to both age-matched control and diuresis (40). Diabetes and diuresis both resulted in significant increases in bladder weight; however, the increase in oxidative stress was specific only to the diabetic model. In another study, diabetic Sprague Dawley rats exhibited a reduction in catalase-like activity, a measure of antioxidant scavenging capability in aerobic cells compared to age-matched control and diuresis (30). Further, these diabetic bladders presented higher levels of apoptotic cells, an increase in lipid peroxidation, and an increase in inducible nitric oxide synthase expression and concluded that oxidative damage to the smooth muscle layer may contribute to DBD symptoms (30). In addition, diabetic bladders from 2 month old rats exhibited increased expression of genes involved in the production or regulation of ROS, higher levels of lipid peroxidation, reduced glutathione S-transferase activity, an increase in protein oxidation and nitrosylation, and increased apoptosis markers (116). Results from this study suggest that increased oxidative stress and apoptosis are associated with impaired bladder contractile capacity. Likewise, bladders from diabetic New Zealand rabbits, exhibited significantly increased lipid peroxides, aldose reductase, and sorbitol compared to both age-matched control and a diuresis model (31).

ROS can also lead to the breakdown of proteins via autophagy, which is a compensatory mechanism for an organism under oxidative stress to preserve cell organelles and proteins. ROS can induce autophagy by oxidizing a cysteine residue in Atg4, which acts to convert microtubule-associated protein light chain 3 to its active form, leading to autophagy (117, 118). A study involving both diabetic human and animal studies showed increased levels of autophagy in β-cells, and further proposed that autophagy serves a protective role against apoptosis by preserving cells and preventing further oxidative damage (119–121). However, persistent autophagy is detrimental as it leads to autophagic cell death (120, 122–124). Consequently, ROS-induced autophagy caused by diabetes can lead to further oxidative stress that damages or destroys smooth muscle cells, contributing to hypocontractility and bladder dysfunction (116).

**Figure 5:**
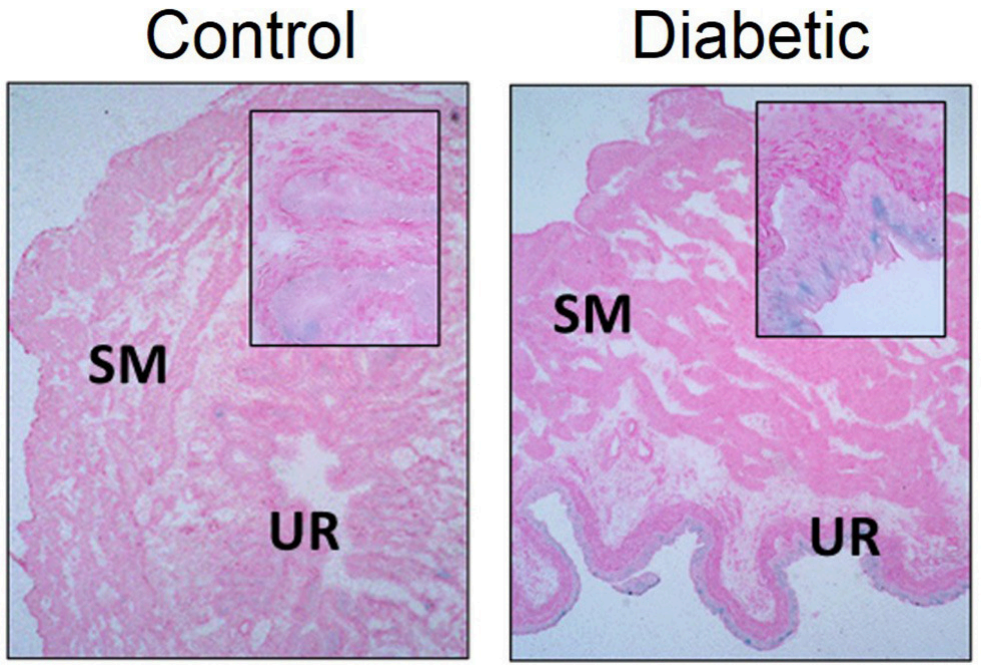
Senescence associated-β-galactosidase staining is increased in diabetic urothelium compared to control counterparts. Senescence associated-β-galactosidase (blue) staining and nuclear fast red counterstain in control (Left) and diabetic (Right) bladders. Diabetes was induced using the combination of a high fat diet and one streptozotocin injection (30 mg/kg) in Wistar animals. Representative images of bladder senescence associated-β-galactosidase staining at 100X, inserts at 400X magnification. SM, smooth muscle. UR, urothelium.

We have observed increased senescence in DBD from type 2 diabetic female Wistar rats (high fat diet and 30 mg/kg i.p. STZ) at 1 month post-STZ injections (Figure 5; original data). At this time point, senescence was localized mainly to the urothelium layer of the bladder, which corresponds with Hanna-Mitchell's findings that the urothelial layer changes both in cell morphology and gene expression (47). Further, gene expression alterations in the diabetic urothelium reported by Hanna-Mitchell et al. are also factors that are altered in senescence (125–127). While inappropriate immune system activation has been well established in diabetes and DBD, it is not known whether the presence of senescent cells can exacerbate the pro-inflammatory/pro-oxidative milieu, thereby worsening LUTS.

## Hypothesis

**Figure 6:**
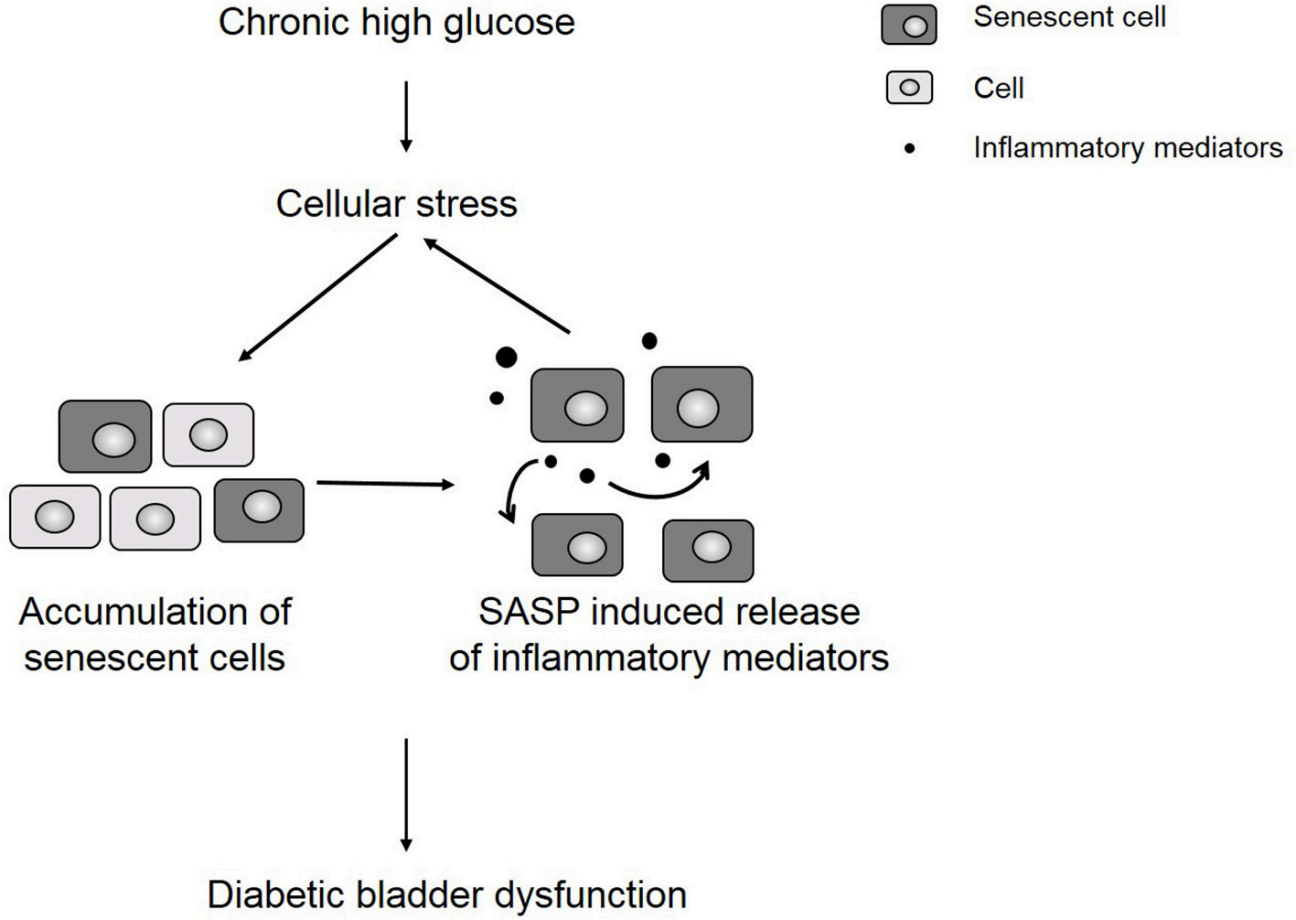
Chronic hyperglycemia contributes to cellular stress and an accumulation of senescent cells. The presence of senescent cells further contributes to increased inflammation in the bladder wall, which leads to symptoms of diabetic bladder dysfunction.

We hypothesize that uncontrolled urothelial senescence contributes to increased inflammation and oxidative stress within the bladder wall and contributes to symptoms of DBD (Figure 6). The SASP of senescent bladder cells, through uncontrolled and excessive pro-inflammatory mediators (e.g., cytokines, chemokines) and ROS generation, could further impair the function of healthy bladder cells, as well as mediate additional transition to the senescent state.

Mechanisms driving initial senescence induction on the apical side of urothelial cells may include chronic increases in urine volume and therefore increased workload and bladder stretch, and exposure to an altered urine content (increased protein, glucose, damaged tubule cell contents). Diabetic kidney disease is a prominent complication of diabetes that is characterized by glomerular hyperfiltration (128). The ultra-filtrate (urine), containing proteins, growth factors, and cytokines, causes apical tubular cell injury and interstitial fibrogenesis within the kidney (128). This ultra-filtrate is transferred from the kidney to the urinary bladder via ureters (128). Therefore, these same factors, which mediated kidney damage, are now exposed to the urothelial layer of the bladder. Increased exposure to chronic high blood glucose concentrations may also affect urothelial cells from the basal side. High and chronic levels of glucose is known to cause mitochondrial dysfunction and increases the expression of advanced glycation end products and ROS, both of which have been shown to induce senescence (129–134).

As mentioned previously, senescent cells release multiple factors (cytokines, ROS, etc.), which may induce DBD symptoms and further drive the accumulation of senescent cells. Oxidative stress, which has been shown in numerous *in vitro* and *in vivo* studies to be remarkably high in diabetic patients, is also a major factor in the activation of cellular senescence (80). The major source of senescence-inducing ROS appears to occur through mitochondrial dysfunction (132), although other enzymatic sources of ROS are also known to contribute (134). Furthermore, increased advanced glycation end products and chronic inflammation, both linked to diabetes, have also been shown to induce senescence (130, 131, 133). Whether accumulating senescent cells could be responsible for or the result of increased inflammation and ROS reported in diabetic bladders is not explicitly known; however, this process could be complementary in that high glucose results in a heightened inflammatory state that increases the accumulation of senescent cells. The SASP could enhance development and accumulation of senescence leading to a positive feedback loop (Figure 3).

The authors believe that the hypothesis presented is testable within the framework of current knowledge. While we have shown increased senescence associated-β-galactosidase staining within the urothelial layer of diabetic bladders, other markers indicative of cellular senescence at different and longer time points and within different models of diabetes should be measured. Multiple standard measurements have been established to indicate senescence. Senescent cells exhibit increased expression of senescence associated-β-galactosidase and p16^INK4A^ as well as secreted inflammatory factors (IL-1, IL-6, IL-8, VEGFA, MMPs) as part of their SASP, measureable by senescence associated-β-galactosidase assays, western blotting, and ELISA (135). Further, increased DNA damage and telomere shortening are also indicators of cellular senescence. The effect and presence of senescence during each state of DBD needs to be investigated coinciding with voiding behavior and cytometric measurements. For example, senescent cells may increase initially after initial insult of DBD due to increased volume of urine and subside with the recruitment of immune cells to the site of injury. One would expect that senescent cells may accumulate again within the later stages of DBD due to increased ROS after prolonged hyperglycemia and chronic increased voiding volume; this would concur with current literature showing the presence of cellular senescence with age as well as enhanced inflammation. One may further postulate if increased urothelial senescence could then lead to increased cellular senescence within the smooth muscle layer of the bladder. There are multiple models of diabetes and all could be used to test this hypothesis. A limitation to the use of diabetic animal models is that these models develop overt disease in short time frames whereas the human condition may develop over longer periods of time and are then managed with various therapeutics and lifestyle interventions; therefore, timing of disease progression and corresponding mechanistic alterations may not be identical. An important control group to consider would be cases of bladder dysfunction, both overactive and underactive bladder, that are not associated with increased blood glucose levels such as diabetes insipidus and bladder dysfunction associated with old age and other disease states. This would determine whether increased cellular senescence was the direct consequence of oxidative stress due to increased and chronic levels of blood glucose. Once evidence of senescence in animal models is established, it would be imperative to show increased urothelial senescence in human DBD patients within both overactive and underactive bladder symptoms possibly through urothelial biopsies.

Some potential limitations to this hypothesis include understanding whether senescent cells are the cause or the effect of DBD *in vivo*. Therefore, although therapeutic targeting of senescent cells may offer symptomatic relief to LUTS, it may not reveal the underlying pathogenic mechanism of bladder dysfunction. Furthermore, parsing out the contribution of senescent urothelial cells to the omnipresent oxidative and inflammatory burden in diabetes could prove challenging. This is especially pertinent given that the specific enzymatic and pattern recognition receptor specific sources of ROS and cytokines, respectively, have not been determined in senescent urothelial cells and will require careful experimental design and potentially novel models of DBD.

## Clinical Perspectives

Due to the insidious onset of DBD, patients may not notice changes in voiding habits until later stages (86). Additionally, patients may not voice their concerns to their healthcare providers unless questioned specifically on symptoms due to the stigma associated with LUTS, particularly with urinary incontinence (136, 137). In one study of women ages 20–45, only 10.8% with overactive bladder and 12.7% with urge incontinence have seen their physicians for those symptoms (136, 138).

Patients with suspected DBD should be evaluated with detailed history and physical exam. Once more serious causes of LUTS are ruled out, patients may undergo urodynamic testing to assess lower urinary tract function (139, 140). Urodynamic testing may not be necessary if diagnosis is clear (139, 140).

Current treatment for DBD include behavioral changes (i.e., weight loss, timed voids, pelvic floor exercises), pharmacological, and surgical interventions (86, 139). For overactive bladder, medications target bladder specific muscarinic receptors (anticholinergics) or ß_3_-adrenergic receptor agonists to reduce detrusor contractions (86, 139). Muscarinic antagonism improves some lower urinary symptoms; however, efficacy is questionable and tolerability is poor due to side effects, the major one being “dry mouth.” In a study of patients with overactive bladder, oxybutynin and tolterodine treatment only resulted in one fewer leakage episode or micturition event per 48 h compared to placebo (141). Similarly, in a study where women took anti-muscarinic agents reported an average of three leaks a day after 6 months of treatment compared to an average of five a day at the start of treatment. Not only is efficacy of these drugs in question, there are significant side effects of these medications such as dry mouth, constipation, difficulty in urination, blurred vision, dry eyes, drowsiness, dizziness, and cognitive decline, which makes long term treatment a challenge for patients (141). The most bothersome side effect for patients is dry mouth; 25% of patients will cease taking this medication because of this side effect. Increased duration of dry mouth will cause dental caries and difficulty in speech; in order to alleviate the dry mouth symptoms, patients will drink more water and therefore exacerbate lower urinary tract symptoms. Patients that fail behavioral modification and pharmacological therapy may benefit from more invasive methods such as intradetrusor botulinum toxin A injections or neuromodulation (86, 139). For underactive bladder, behavior modifications are encouraged but pharmacologic options are limited; current treatment options include methods such as asking patients to “double-void,” placement of an indwelling catheter, and intermittent self-catheterization (86). Long-term use of indwelling catheters are associated with high risk of complications such as urinary tract infections and urethral lesions (142, 143). Ultimately, other surgical intervention may be explored for both overactive and underactive bladder if the patient does not respond to less invasive treatment options (86, 139).

## Senescence as a Novel Target for Diabetic Bladder Dysfunction Treatment

Aged or diseased tissues are not able to efficiently clear senescent cells, thereby resulting in their accumulation (64). Therefore, it is not surprising that recent studies support the therapeutic approach of targeting senescence for the treatment (144–146). Agents that prevent the activation of specific mechanisms of senescence, such as those involving telomerase, DNA-damage repair machinery, cell-cycle checkpoint kinases, and tumor suppressors, are all known to reduce indices of disease (69, 147). Similarly, the removal of senescent cells was recently shown to delay the onset of several age-related disease processes (72, 144).

As mentioned previously, LUTS is seen in up to half of diabetic patients and is associated with a significant negative impact on quality of life. With the increasing prevalence of diabetes, it is expected that complication of diabetes, such as DBD, is also on the rise. Further investigation into the association between urothelial senescence and DBD may lead to new therapeutic pathways. Senolytic therapy during the early stages of DBD may prevent further oxidative stress and possibly prevent progression of early stages of DBD to a later stage.

Agents that specifically target senescent cells for apoptosis represents a promising field of pharmacology and could be a more selective option for the treatment of DBD. To illustrate the impact of senescent cells on its surrounding tissue, one study showed that transplanting senescent ear chondrocytes into the knee joint region caused osteoarthritis-like condition in mice, while transplanting non-senescent cells did not (148, 149). A recent study reported that administration of two senolytics, dasatinib, and quercetin, reduced physical dysfunction induced by senescent cell transplantation and increased lifespan in old mice (149). Currently, senolytic therapy is used in some cancer treatments. For example, dasatinib is a senolytic used to treat certain forms of acute lymphoblastic leukemia and chronic myeloid leukemia (150). Other senolytics, such as navitoclax and UBX0101, are under clinical trials for the treatment of solid tumors and osteoarthritis, respectively. A recent article highlights the use of FOXO4-p53 interfering peptide, causing p53 nuclear exclusion in senescent cells and apoptosis, and resulting in protection against chemotoxicity and restoring the effects of age (151). Apoptosis of bladder senescent cells could decrease hypertrophy and prevent increased ROS and inflammation, both of which are characteristic of DBD. Interestingly, metformin is being evaluated as a SASP regulator for its effects on inhibiting the NF-κB pathway, ROS production, and inflammation (152, 153). The effects of metformin on urothelial senescence would be hard to elucidate; not only would metformin decrease plasma and urine glucose levels, it also acts as a SASP regulator. Further, treatment with sodium-glucose co-transporter-2 inhibitors would be an interesting positive control if increased urine glucose levels are responsible for increased urothelial senescence (154).

## Conclusion

Despite recent advances in understanding DBD, the underlying pathways are poorly understood and therapeutic options are limited and not always effective, highlighting the need for novel interventions (13, 22). While cellular senescence is a normal physiological response to stress, uncontrolled senescence can be detrimental to human health in high accumulating numbers as evidenced in multiple age-related disease states, including diabetes. Numerous studies suggest that the increased presence of senescent cells leads to tissue dysfunction. In addition, urothelial barrier dysfunction is associated with symptoms of urgency and frequency. Therefore, we put forward the hypothesis that increased senescent urothelial cells in diabetic bladders contribute to symptoms of DBD. Because the urothelium plays an important role not only as a barrier to urine, but also as a sensor, identifying molecular mechanisms leading to senescence is essential. Additional work is needed to link the clinical symptoms of each state of DBD with the presence of cellular senescence within urinary bladder layers. Normal aging induced senescence and possible bladder dysfunctions associated with the aging process, as well as sex, hormonal status, diabetes duration, and reversal with treatment/exercise need to be investigated in coordination with bladder cellular senescence and DBD.

## Ethics Statement

All animal procedures were performed in accordance with the Guide for the Care and Use of Laboratory Animals from the National Institutes of Health and were reviewed and approved by the Institutional Animal Care and Use Committee of Augusta University.

## Author Contributions

NK designed the hypothesis, conducted experiments, and wrote the paper. CM contributed to and edited the paper. JV contributed to the initial research into the inflammation and oxidative stress section. SL researched, edited, and rewrote the section. JM wrote the clinical perspectives section, contributed to the section senescence as a novel target for diabetic bladder dysfunction treatment, and edited the paper. RW designed the hypothesis and edited the paper.

### Conflict of Interest Statement

The authors declare that the research was conducted in the absence of any commercial or financial relationships that could be construed as a potential conflict of interest.

## Abbreviations

ATP: adenosine triphosphate
DBD: diabetic bladder dysfunction
CDK: cyclin-dependent kinase
COX: cyclooxygenase
GI: gastrointestinal
IC: interstitial cells
ICC: interstitial cells of Cajal
IL: interleukin
LUTS: lower urinary tract symptoms
M: muscarinic receptor
MMP: matrix metalloproteinases
MYD88: myeloid differentiation primary response 88
NF-κB: nuclear factor-kappa B
ROS: reactive oxygen species
SASP: senescence-associated secretory phenotype
SHH: sonic hedgehog
STZ: streptozotocin
TLR: toll-like receptors
TNF: tumor necrosis factor
VEGFA: vascular endothelial growth factor A
WNT: wingless-related integration site.
